# Disruption in iron homeostasis and impaired activity of iron-sulfur cluster containing proteins in the yeast model of Shwachman-Diamond syndrome

**DOI:** 10.1186/s13578-020-00468-2

**Published:** 2020-09-11

**Authors:** Ayushi Jain, Phubed Nilatawong, Narinrat Mamak, Laran T. Jensen, Amornrat Naranuntarat Jensen

**Affiliations:** 1 Department of Pathobiology, Faculty of Science, Mahidol University, 272 Rama 6 Road, Bangkok, 10400 Thailand; 2 Division of Biopharmacy, Faculty of Pharmaceutical Sciences, Ubon Ratchathani University, Ubon Ratchathani, 34190 Thailand; 3 Toxicology Graduate Program, Faculty of Science, Mahidol University, Bangkok, 10400 Thailand; 4 Department of Biochemistry, Faculty of Science, Mahidol University, Bangkok, 10400 Thailand; 5 Pathology Information and Learning Center, Department of Pathobiology, Faculty of Science, Mahidol University, Bangkok, 10400 Thailand; 6 Center of Excellence on Environmental Health and Toxicology (EHT), Bangkok, Thailand

**Keywords:** Shwachman-diamond syndrome, Iron overload, Yeast, Iron sulfur cluster, *POR1* overexpression

## Abstract

**Background:**

Shwachman-Diamond syndrome (SDS) is a congenital disease that affects the bone marrow, skeletal system, and pancreas. The majority of patients with SDS have mutations in the *SBDS* gene, involved in ribosome biogenesis as well as other processes. A *Saccharomyces cerevisiae* model of SDS, lacking Sdo1p the yeast orthologue of SBDS, was utilized to better understand the molecular pathogenesis in the development of this disease.

**Results:**

Deletion of *SDO1* resulted in a three-fold over-accumulation of intracellular iron. Phenotypes associated with impaired iron-sulfur (ISC) assembly, up-regulation of the high affinity iron uptake pathway, and reduced activities of ISC containing enzymes aconitase and succinate dehydrogenase, were observed in *sdo1*∆ yeast. In cells lacking Sdo1p, elevated levels of reactive oxygen species (ROS) and protein oxidation were reduced with iron chelation, using a cell impermeable iron chelator. In addition, the low activity of manganese superoxide dismutase (Sod2p) seen in *sdo1*∆ cells was improved with iron chelation, consistent with the presence of reactive iron from the ISC assembly pathway. In yeast lacking Sdo1p, the mitochondrial voltage-dependent anion channel (VDAC) Por1p is over-expressed and its deletion limits iron accumulation and increases activity of aconitase and succinate dehydrogenase.

**Conclusions:**

We propose that oxidative stress from *POR1* over-expression, resulting in impaired activity of ISC containing proteins and disruptions in iron homeostasis, may play a role in disease pathogenesis in SDS patients.

## Background

Shwachman-Diamond syndrome (SDS) is an autosomal recessive disorder characterized as a ribosomopathy [1–4]. Defects in ribosome biogenesis, due to mutations in ribosomal proteins and assembly factors, can lead to a wide range of clinical phenotypes [5]. Patients with SDS suffer from bone marrow failure, exocrine pancreatic dysfunction, skeletal abnormalities, and have a high risk of malignant transformation [6–11]. Even though SDS is a rare genetic disorder, it is a common cause of inherited exocrine pancreatic dysfunction and bone marrow failure [12–15].

The majority of SDS patients have loss of function mutations in SBDS (SDS1, OMIM #260400) [16]. The best characterized function of SBDS is its role in the release and recycling of eIF6 from pre-60S ribosomes, a process that also involves the GTPase activity of Elongation Factor-Like 1 (EFL1) (SDS2, OMIM # 617941) [17–20]. The release of eIF6 is a required step in the maturation of the 60S ribosomal subunit [1, 3, 21, 22]. Reduced formation of mature 60S ribosome subunits results in impaired protein translation in cells with SDS deficiency [3, 23, 24]. SBDS physically interacts with EFL1 and mutations in EFL1 are also associated with SDS [17–20]. Interestingly, several SBDS missense mutations reduce binding affinity with EFL1 [18]. In addition, clinical features similar to SDS have been reported for mutations in DnaJ Heat Shock Protein Family (Hsp40) Member C21 (DNAJC21, OMIM # 617052), a protein that may act as a co-chaperone for HSP70 [25, 26] and the Signal Recognition Particle 54 (SRP54, OMIM # 618752), that binds to the signal sequence of presecretory proteins when they emerge from the ribosomes and directs them to the translocon on the endoplasmic reticulum membrane [27, 28].

SBDS is a multi-functional protein and SBDS mutations impact processes beyond ribosome biogenesis. Additional proposed functions for SBDS include rRNA processing [22, 29, 30], cellular stress responses [31, 32], and lysosome function [33]. Cells depleted for SBDS also appear to be under a chronic state of stress [34–36] and are sensitized to agents that impair endoplasmic reticulum (ER) function and conditions that promote DNA damage [31]. Mammalian cells deficient for SBDS and yeast cells lacking Sdo1p, the orthologue of SBDS, also display sensitivity to oxidative and osmotic stress [32, 37]. Yeast deleted for *SDO1* exhibit mitochondrial dysfunction, instability of mtDNA, and decreased activity of mitochondrial manganese superoxide dismutase (Sod2p) [32, 38, 39]. Stress sensitivity and mitochondrial dysfunction in cells deficient for SBDS/Sdo1p appears to be separate from attenuated protein translation [31, 32, 39].

Even with better understanding of the functions of SBDS/Sdo1p, the molecular mechanisms of how mutations in SBDS impact disease progression remains unclear. Current treatments for SDS patients do not target the underlying cause of the disease and instead are aimed at alleviating symptoms of this disorder. Elucidation of the key steps in the cellular pathogenesis of SDS should enable the development of therapies capable of targeting deficiencies leading to the disease state.

Using a *Saccharomyces cerevisiae* model of SDS, we examined potential causes of cellular stress from impaired Sdo1p function. Oxidative damage and inactivation of Sod2p in *sdo1*∆ cells previously described [39] were similar to effects seen in yeast mutants that over-accumulate intracellular iron due to defects in iron sulfur cluster (ISC) biogenesis [40, 41]. Examination of cellular iron content demonstrated that *sdo1*∆ cells accumulate three-fold more iron compared to wild-type yeast. Limiting iron accumulation in *sdo1*∆ cells reduced oxidative damage and environmental stress sensitivity as well as rescuing low activity of Sod2p. However, impaired activity of ISC enzymes in *sdo1*∆ cells was not restored with iron chelation. Deletion of *POR1* is known to alleviate stress sensitivity in *sdo1*∆ cells [32] and we report that iron accumulation is also reduced in *por1*∆ *sdo1*∆ yeast. In addition, activity of ISC enzymes in *sdo1*∆ cells was improved by prior deletion of *POR1*. Our results indicate that loss of Sdo1p is linked to disruptions in ISC biogenesis, mediated by over-expression *POR1*, that potentially promotes subsequent iron over-accumulation.

## Results

### Cells deficient for Sdo1p display elevated iron content

**Fig. 1.**
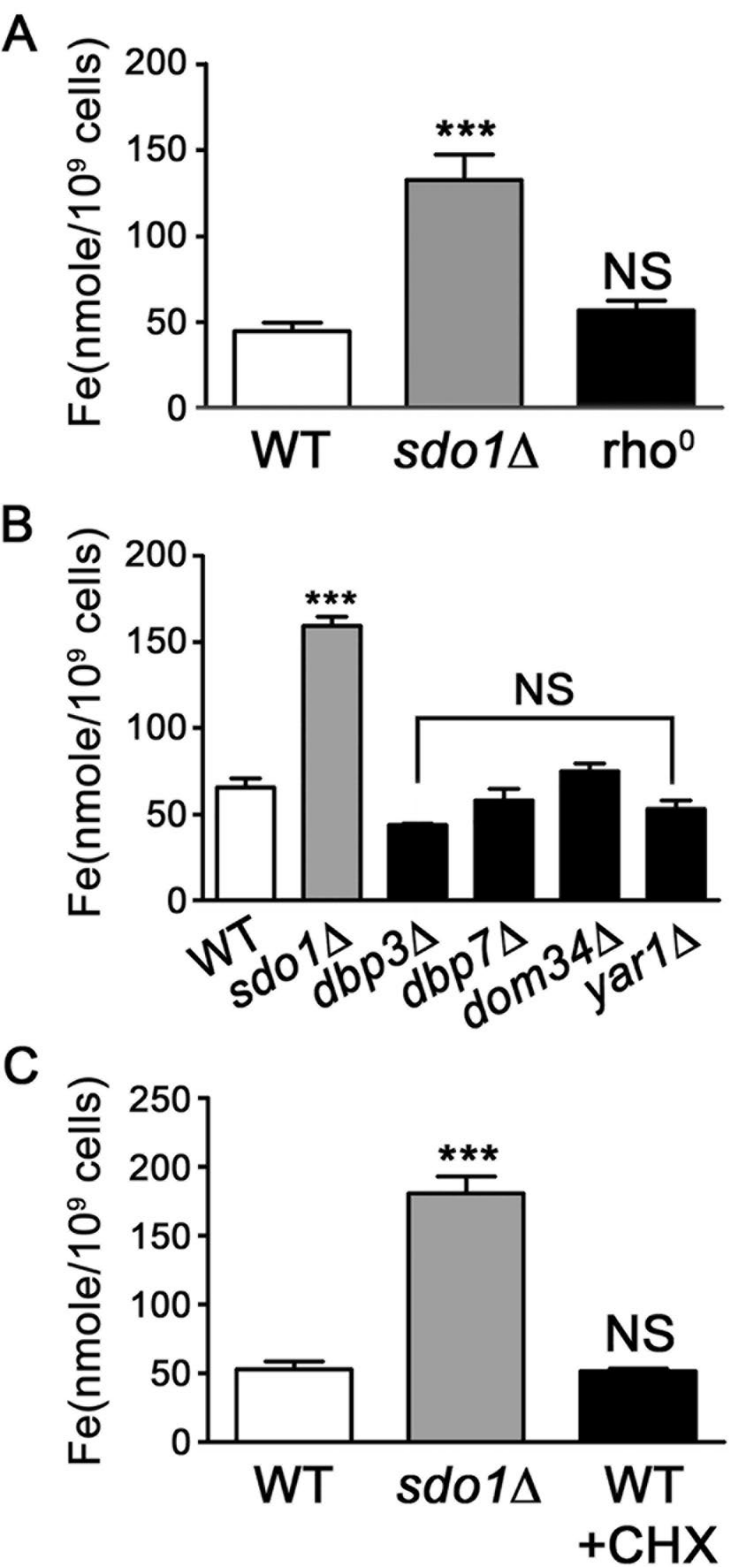
Over-accumulation of intracellular iron was observed in cells deficient for Sdo1p. **a**, **b** Wild type (WT), *sdo1*Δ, rho^0^, and ribosome defective mutants (*dbp3∆, dbp7∆, dom34∆* and *yar1∆*) were cultured overnight in liquid YPD medium and the intracellular iron content was measured using graphite furnace atomic absorption spectrometry. Data shown are from three independent experiments. **c** Yeast were treated with 0.1 mg/mL cycloheximide (CHX) for 15 minutes to inhibit translation, intracellular iron levels were measured as described above. Data shown are from three independent experiments. Values are mean + SD, ***P <0.001, NS: not significant, determined using Student's T-test compared to WT.

Our previous studies reported elevated oxidative stress as well as reduced levels and activity of manganese-containing superoxide dismutase 2 (Sod2p) in yeast cells lacking Sdo1p [32, 39]. Iron released from the ISC biogenesis pathway is highly reactive and can inactivate Sod2p through competition with manganese binding to this enzyme [40, 41]. We therefore investigated whether iron homeostasis is altered in *sdo1*∆ yeast. Cells lacking Sdo1p accumulated three-fold higher levels of iron compared to wild-type yeast (Fig. 1a), similar to that seen for several yeast mutants with defects in ISC biogenesis [42–47]. The elevated iron content of *sdo1*∆ cells does not appear to be associated with loss of mitochondrial DNA (mtDNA), as iron content in a rho^0^ strain lacking mtDNA was similar to WT yeast (Fig. 1a).

Reduced translational efficiency observed in *sdo1*∆ cells [1, 2], was not associated with iron over-accumulation. Yeast mutants that exhibit reduced polysome numbers: *dbp3∆, dbp7∆, dom34∆* and *yar1∆* [48–51] did not exhibited increased iron content relative to WT cells (Fig. 1b). Similarly, limiting protein synthesis in WT cells using cycloheximide did not result in elevated iron accumulation (Fig. 1c). These results indicate that iron over-accumulation in cells lacking Sdo1p is not directly linked to the reduced translational efficiency.

### Depletion of iron improves the activity of manganese-superoxide dismutase Sod2p and reduces oxidative stress in cells lacking Sdo1p

**Fig. 2.**
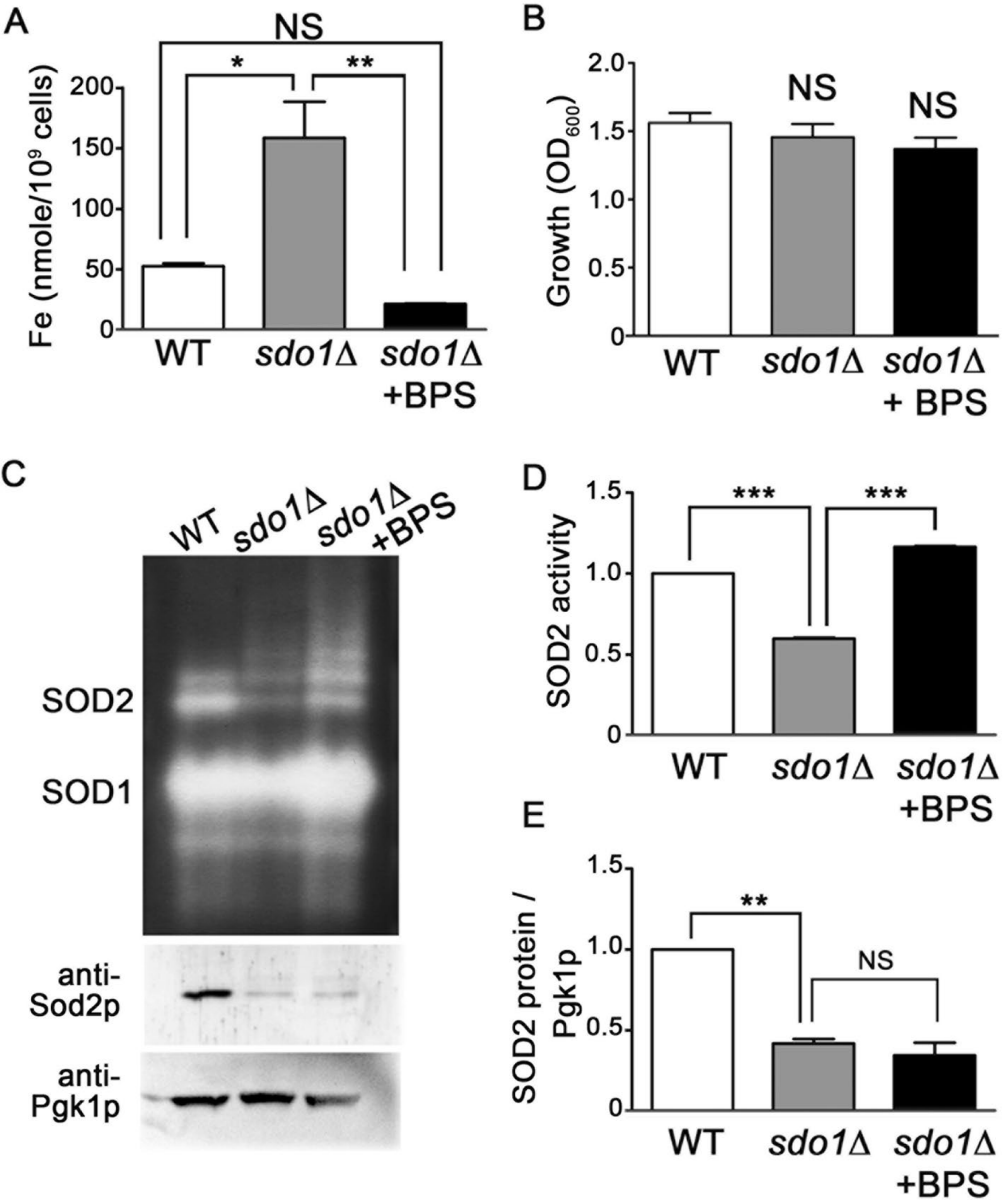
Iron depletion enhances Sod2p activity in *sdo1*∆ cells. The designated strains were cultured in YPD medium containing where indicated (+BPS) 40 μM BPS. **a** The intracellular iron levels were examined as in Fig. 1. Data shown are from three independent experiments. **b** Growth was monitored by monitoring OD 600 nm at 21 hours. **c** Whole-cell lysates were analyzed for SOD activity by native gel electrophoresis and nitroblue tetrazolium staining. SOD1 and SOD2 indicate positions of active Cu/Zn-containing Sod1p and manganese-containing Sod2p respectively. Sod2p and Pgk1p polypeptides from the whole-cell lysates were analyzed by immunoblot (bottom panel). **d** Sod2p activity was quantitated and expressed as ratio of Sod2p activity/Pgk1p for each sample, normalized to WT = 1. **e** Quantitation of Sod2p protein levels normalized to Pgk1p abundance in each sample, with WT = 1. Results are from two independent experiments. Values are the mean + SD. ***P <0.001, **P <0.01, *P <0.05, and NS (not significant) determined using Student’s T-test compared between the means of two indicated groups.

We speculated that iron over-accumulation may contribute to the lowered levels of Sod2p activity in *sdo1*∆ cells. Treatment with the cell impermeable iron chelator, bathophenanthroline disulfonic acid (BPS), significantly reduced iron content of *sdo1*∆ cells (Fig. 2a), without altering growth rates (Fig. 2b). As seen in Fig. 2c, d, Sod2p activity was increased in iron depleted *sdo1*∆ cells, suggesting that the excess iron in *sdo1*∆ can bind and inactivate Sod2p. However, following iron depletion the abundance of Sod2p protein in *sdo1*∆ yeast remained low compared to WT cells (Fig. 2c and e). This indicates that the proposed iron incorporation into Sod2p only accounts for a portion of the loss of Sod2p activity in *sdo1*∆ cells.

**Fig. 3.**
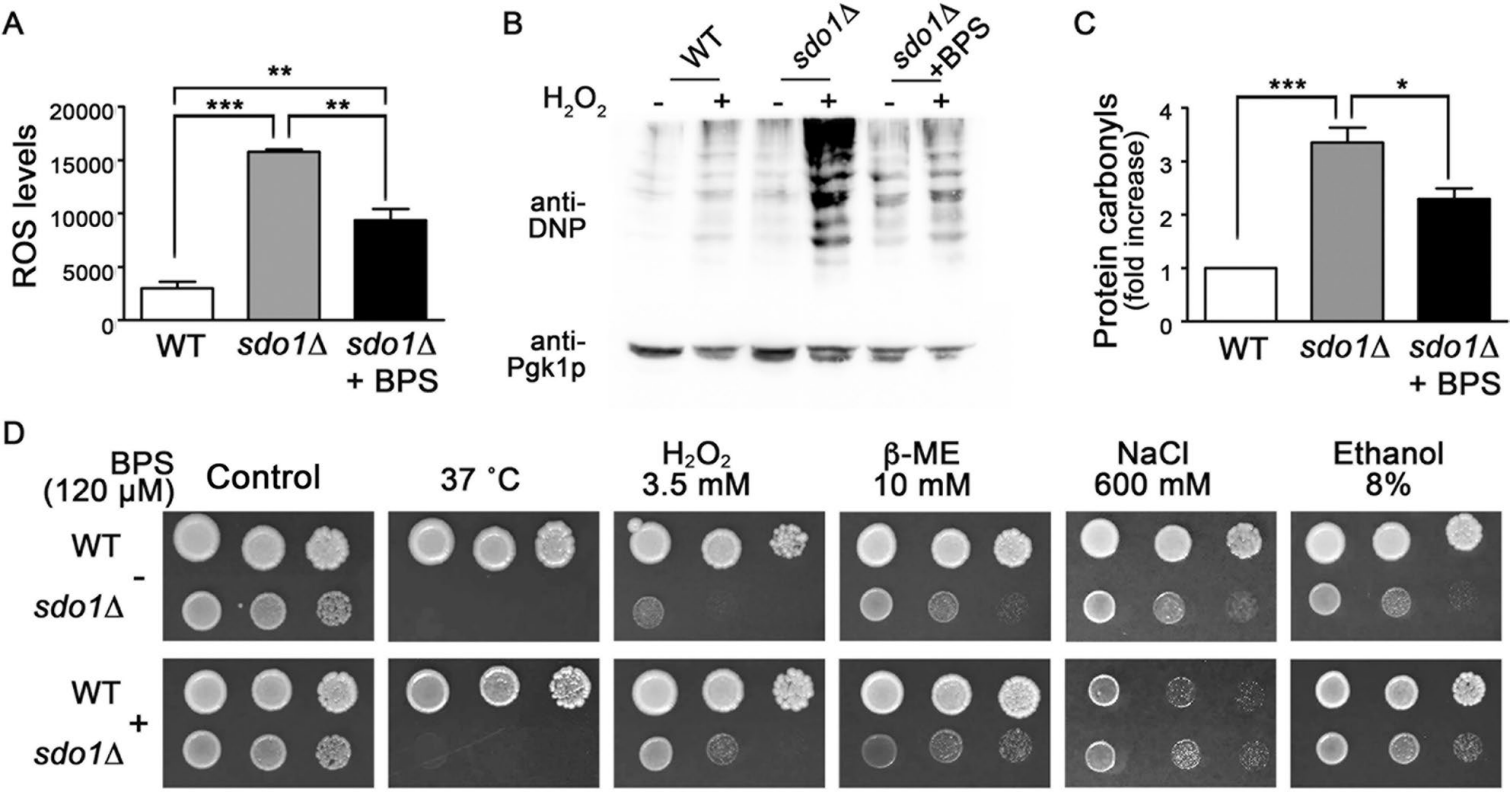
ROS production, protein oxidation, and H_2_O_2_ sensitivity are reduced in *sdo1*∆ yeast with iron depletion. **a** Intracellular ROS levels were monitored using DCFH-DA. The fluorescence intensity of each sample was normalized to protein content. Data shown are from three independent experiments. **b** Protein carbonyls were determined in whole cell lysates prepared from indicated strains untreated (H_2_O_2_: -) or treated with 2 mmol/L H_2_O_2_ (H_2_O_2_: +). DNPH-reactive carbonyl groups were detected by immunoblotting with an anti-DNP antibody. Immunostaining of phosphoglycerol kinase (Pgk1p) was used as loading control. **c** Quantitation of protein carbonylation results normalized to the intensity of Pgk1p with levels normalized to WT = 1. Data were from three independent experiments. **d** Growth of WT and *sdo1*∆ cells was monitored by spotting 10^4^, 10^3^, and 10^2^ cells onto solid YPD medium alone (control) or supplemented with 120 µM BPS. The indicated concentrations of stressors were also included. Plates were incubated at 30 ˚C for 3 days and photographed. For heat stress, cells were incubated at 37 ˚C. Data are representative of three independent experiments. Values are the mean + SD. ***P < 0.001, **P <0.01, and *P < 0.05, determined using Student’s T-test compared between the means of two indicated groups.

Consistent with this observation, ROS levels in cells lacking Sdo1p were also significantly reduced when iron content was limited by growth with BPS; however, ROS production remained elevated compared to cells with intact Sdo1p (Fig. 3a). Protein oxidation from H_2_O_2_ exposure was also significantly decreased in cells lacking Sdo1p when intracellular iron was depleted (Fig. 3b and c). Limiting iron with BPS was capable of enhancing growth of *sdo1*∆ cells exposed to H_2_O_2_ (3.5 mM) and to a lesser extent 8% ethanol (Fig. 3d). However, iron depletion did not alleviate slow growth of *sdo1*∆ yeast under conditions of heat stress (37 ˚C), reductive stress (10 mM β-ME), or salt stress (600 mM NaCl). It appears that the excess iron present in cells deficient for Sdo1p contributes to the elevated oxidative stress and damage seen in this strain with lowered Sod2p activity likely aggravating this effect.

### Mis-regulation of iron uptake and impaired activities of ISC enzymes in cells deficient for Sdo1p

**Fig. 4.**
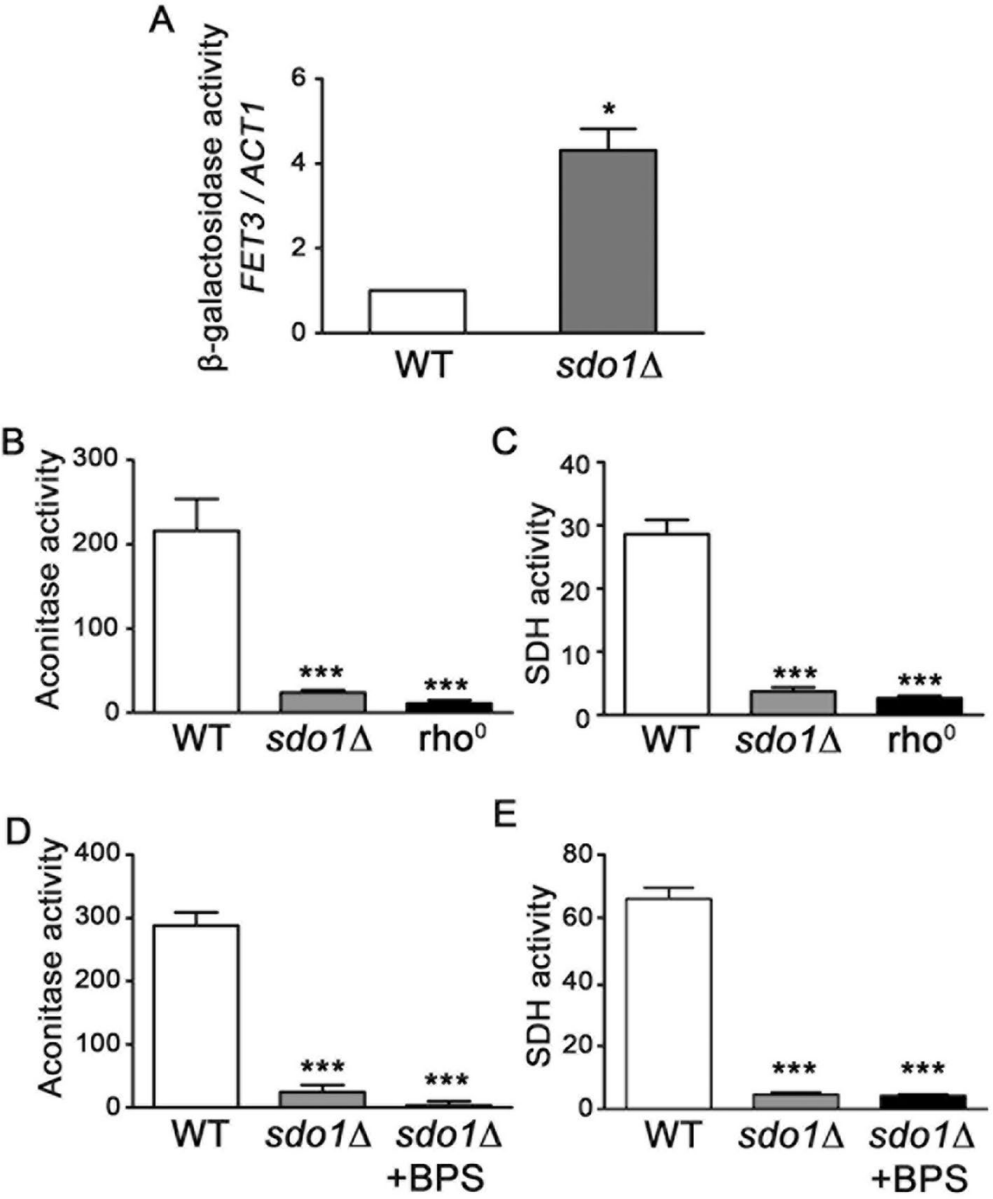
Cells lacking Sdo1p show a reduction in ISC enzyme activities. **a** The induction of *FET3* utilizing *FET3-lacZ* reporter plasmid containing the *lacZ* gene under control of the *FET3* element was monitored. WT and *sdo1*∆ cells were transformed with the plasmid pLJ440 (*FET3-lacZ*). Transformed cells were cultured in SC-URA selective media for 21 hours at 30°C until the OD_600_ of approximately 1.2. Data shown are from three independent experiments. **b** Aconitase activity was measured using whole cell lysates of WT, *sdo1*∆ and rho^0^ cells. The cells were cultured in YPD media with 2% glucose as a carbon source for 40 hours in order to reach stationary phase following by extraction of proteins to determine the activity. Data shown are from three independent experiments. **c** Succinate dehydrogenase (SDH) activity was measured in WT, *sdo1*∆ and rho^0^ cells. The cells were cultured in YPD media with 2% glucose as a carbon source for 40 hours in order to reach stationary phase following by mitochondrial isolation and protein extraction for determining the SHD activity. Data shown are from three independent experiments. **d** and **e** Activities of aconitase and SDH were monitored in WT, *sdo1*∆ and *sdo1*∆ strain treated with BPS. BPS was added to the media 24 hours before culture. Cells were cultured and the activities of aconitase and SDH were analyzed as described in **b** and **c** respectively. Data shown are from three independent experiments. Values are the mean + SD. ***P < 0.001 and *P < 0.05, determined using Student’s T-test compared  to WT.

Defects in ISC formation lead to mis-regulation of iron uptake and iron over-accumulation [42, 43, 52, 53]. The yeast *FET3* gene, encoding a homologue of ceruloplasmin, is required for high affinity iron uptake and its expression is elevated under conditions of ISC deficiency [54, 55]. *FET3* expression, monitored using a *FET3-lacZ* reporter plasmid, was approximately four times higher in *sdo1*∆ cells compared to WT (Fig. 4a), indicating impaired sensing of iron status. The activities of the ISC-containing enzymes aconitase and succinate dehydrogenase are also significantly reduced in the *SDO1* deletion strain, a rho^0^ strain lacking mtDNA was used as a control (Fig. 4b, c). In contrast to the partial rescue of Sod2p activity by iron depletion, treatment with BPS did not enhance activity of aconitase or succinate dehydrogenase (Fig. 4d, e). It appears that cells lacking Sdo1p have defects in ISC containing proteins and this is a likely cause of mis-regulation of iron sensing leading to the increased labile pool of intracellular iron.

### Deletion of *POR1* in *sdo1*∆ cells limits iron over-accumulation and enhances activity of ISC enzymes

**Fig. 5.**
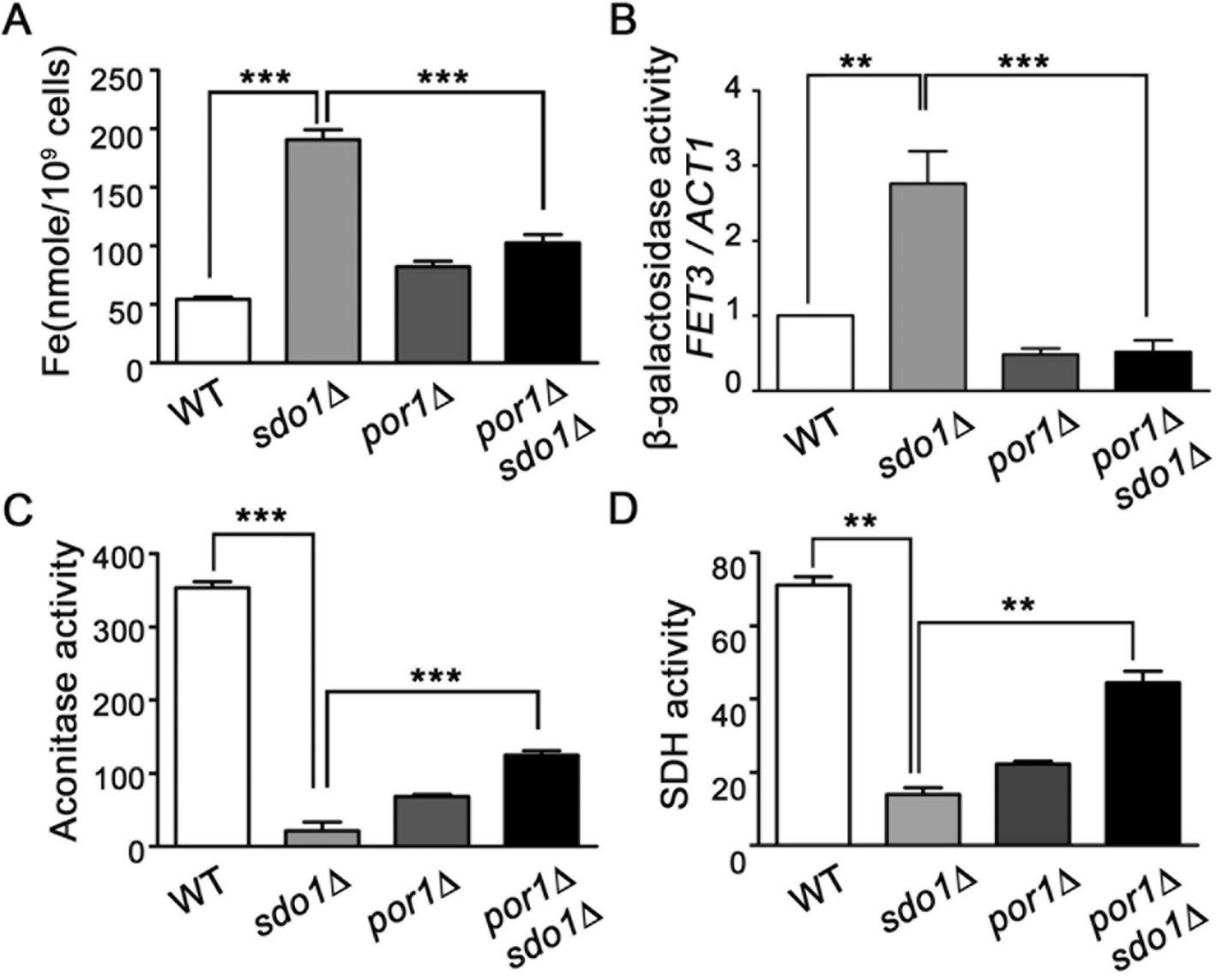
Prior deletion of *POR1* restores defects observed in cells lacking Sdo1p. **a** Intracellular iron levels of WT, *sdo1*∆, *por1*∆ and *sdo1∆por1∆*, as indicated, were examined as described in Fig. 1. Data were from three independent experiments. **b**–**d** The induction of *FET3-lacZ* and activities of aconitase and succinate dehydrogenase were monitored as described in Fig. 4. Indicated strains were cultured in YPD media with 2% glucose for 40 hours until reaching stationary phase. Data were from three independent experiments. Values are the mean + SD. ***P < 0.001 and **P < 0.01 determined using Student’s T-test compared between the means of two indicated groups

Protein levels of Por1p, the yeast orthologue of human mitochondrial outer membrane VDAC, are significantly increased in *sdo1*Δ yeast [32, 38]. We previously demonstrated that prior disruption of *POR1* is able to substantially abrogate the effects of *SDO1* deletion [32], including improving Sod2p activity [39]. Prior deletion of *POR1* is able to significantly reduce iron content of *sdo1*∆ cells (Fig. 5a) and prevents induced expression of *FET3* (Fig. 5b). In addition, increased activity of aconitase and succinate dehydrogenase is observed in *por1∆sdo1∆* cells relative to the *sdo1*∆ strain, although the activity of these enzymes remains low compared to WT yeast (Fig. 5c, d). These data suggest that increased expression of Por1p in *sdo1∆* cells plays a role in loss of enzymatic activity of ISC-containing proteins, promoting mis-regulation of iron uptake and over-accumulation of intracellular iron.

## Discussion

The majority of SDS patients have mutations in the *SBDS* gene, which encodes a protein important for ribosomal maturation [1, 16]. However, SBDS appears to be involved in other cellular pathways including chemotaxis [56], response to stress [31, 36, 57], and cell survival [58, 59]. Even with better understanding of SBDS functions beyond ribosome biogenesis, the molecular mechanisms of how loss of SBDS impacts disease progression remains unclear.

The use of simple model organisms can aid in understanding how disease associated mutations lead to pathogenesis at a cellular level. The yeast model of SDS in which *SDO1*, the yeast orthologue of human *SBDS*, has been deleted has facilitated the identification of additional pathways that require this gene for normal function. Enhanced sensitivity to oxidative stress insults and damage to mitochondria is apparent in yeast lacking Sdo1p [32, 39]. These effects appear to be mediated in part through a significant reduction in activity of Sod2p [39], a key enzyme required for defense against ROS produced in mitochondria [60].

Defects in mitochondrial processing and clearance of Sod2p presequence peptides has been suggested to contribute to reduced Sod2p activity and protein in *sdo1*∆ cells [39]. Additionally, several reports have linked reduced Sod2p activity to over-accumulation of iron, where excess iron competes with manganese for occupancy of the Sod2p active site. These observations are from both yeast disease models and tissues of patients suffering from diseases associated with iron overload, such as Friedrich’s Ataxia and hemochromatosis [40, 41, 61, 62].

Interestingly, expression of transferrin receptor 2 (*TFR2*), involved in iron homeostasis, is upregulated in *SBDS* deficient cells [34]. Over-expression of *TFR2* in cultured human cells leads to increased iron uptake and accumulation [63]. We speculated that elevated iron accumulation may impact SDS progression, potentially through enhancing oxidative stress and interfering with manganese incorporation into Sod2p. Consistent with the reported increase of *TFR2* expression in SDS cells, *sdo1*∆ yeast accumulate three times more intracellular iron compared to cells with intact Sdo1p. Although differences exist in the regulation and use of cellular iron between human and yeast cells, many aspects are similar. Genes involved in ISC assembly and non-heme iron uptake are largely conserved between yeast and human cells [64–66]. The regulation of iron uptake and storage genes is distinct with transcriptional systems present in yeast compared to translational or post-translational regulation in human cells [67, 68]. However, sensing of iron status in both human and yeast utilizes ISCs as the signaling molecule [69, 70].

Our findings indicate that the elevated iron content of *sdo1*∆ yeast is not associated with loss of mitochondrial DNA, defects in ribosome biogenesis, or reduced protein translation. Yeast strains lacking mtDNA or carrying deletions for genes involved in ribosome maturation show similar iron content to wild-type cells. In addition, chemical inhibition of protein translation in wild type yeast cells does not promote iron accumulation. Limiting iron availability, through the use of a cell impermeable iron chelator (BPS) significantly decreased ROS levels in cells lacking Sdo1p. Oxidative damage to proteins following hydrogen peroxide stress was also reduced by iron chelation of *sdo1*∆ cells. It appears that iron mediated formation of ROS is a major cause of oxidative stress in cells lacking Sdo1p.

The active site of Sod2p can bind to both iron and manganese with similar affinities, although binding of iron results in an inactive form of the enzyme [71, 72]. Sod2p activity was enhanced following iron chelation, indicating that reduced activity of this enzyme is mediated in part through inappropriate binding of iron to its active site. Surprisingly, the level of Sod2p protein was not increased with iron chelation. Thus, it seems that the lowered Sod2p protein levels are not due to iron over-accumulation but instead appear to be linked to the impaired import of pre-Sod2p into mitochondria previously reported [39]. However, excess iron further limits the activity of Sod2p in addition to the effect seen from reduced accumulation of the Sod2p protein. Preventing accumulation of excess iron appears to be sufficient to reduce competition between iron and manganese for access to the Sod2p active site in *sdo1*∆ cells, enhancing Sod2p activity.

Distinct pools of iron are present in mitochondria and iron released from ISC proteins or from the ISC biogenesis pathway is highly reactive with Sod2p and can limit the activity of this enzyme [40, 41]. A well-established cellular response to defects in the ISC pathway is increased expression of genes involved in iron uptake [54], including *FET3*, encoding a homologue of ceruloplasmin [73]. In yeast lacking Sdo1p, *FET3* expression was significantly increased relative to wild-type cells, consistent with a defect in the mitochondrial ISC pathway [54, 74, 75]. Significant reductions in the activity of ISC-containing enzymes aconitase and succinate dehydrogenase was also observed in *sdo1*∆ yeast. Iron chelation did not restore the activity of aconitase or succinate dehydrogenase in *sdo1*∆ cells, suggesting that inactivation of these enzymes may not be a direct result of iron-induced oxidative damage. Although it is possible that the loss of ISC enzyme activities in iron-depleted cells is due to the fact that the cells remain lacking of mitochondrial DNA as we can observe loss of similarly loss of ISC enzyme activities in rho^0^ strain. Taken together these observations are consistent with impairment of the ISC pathway, which may be the source of iron over-accumulation due to deletion of *SDO1*.

The connection between loss of Sdo1p and impaired activity of the ISC pathway remains unclear. However, a significant over-expression of *POR1*, encoding a mitochondrial voltage-dependent anion channel (VDAC), has been demonstrated in cells lacking Sdo1p [32, 38]. Over-expression of *POR1* results in oxidative damage to proteins and increased sensitivity to hydrogen peroxide [32]. The impact of loss of Sdo1p on the ISC pathway may be mediated through its effects on Por1p over-accumulation and resulting oxidative stress. The ISC biosynthesis pathway as well as many mature ISC containing enzymes, such as aconitase, are sensitive to ROS [76, 77]. Release of iron from the ISC pathway can contribute to elevated iron content, leading to a further increase in ROS production [41, 46, 78]. In this analysis, prior deletion of *POR1* in *sdo1*∆ strains was found to significantly restore regulation of iron metabolism, lowering cellular iron content, and enhancing the activity of aconitase and succinate dehydrogenase. Prior deletion of *POR1* was previously found to significantly increase Sod2p activity in *sdo1*∆ cells [39], consistent with the results reported here for iron chelation, further indicating a link between elevated Por1p levels and mis-regulation of iron homeostasis.

## Conclusions

The findings from this study have revealed a novel pathway affected by impaired Sdo1p activity. The mechanisms that promote iron over-accumulation and impaired ISC biogenesis in cells lacking Sdo1p remain to be clarified. However, elevated ROS observed in SBDS deficient cells [34] as well as *sdo1*∆ yeast [32] may contribute to cellular damage and dysfunction. ISCs in many proteins are ROS labile and their disassembly can contribute to increased levels of reactive cellular iron [41, 79]. A cycle in which ROS mediated release of iron from ISCs promotes ROS formation may be contributing to cellular damage seen in *sdo1*∆ cells. Impaired activity of Fe-S containing enzymes in the mitochondrial respiratory chain was observed in cells lacking Sdo1p. We propose that these effects are a consequence of elevated ROS production, mediated by *POR1* over-expression. Better understanding of how decreased Sdo1p/SBDS activity contributes to altered activity ISC enzymes and how these effects are associated with SDS pathogenesis, may aid in developing therapeutic strategies for this disease. If iron over-accumulation and impaired activity of ISC enzymes is confirmed in SDS patients, interventions that can alleviate these effects may aid in the management of SDS symptoms.

## Materials and methods

### Yeast strains, culture conditions, and plasmids

The strains utilized in this study are isogenic to haploid strains BY4741 (*MATa*, *leu2*Δ*0*, *met15*Δ*0*, *ura3*Δ*0*, *his3*Δ*1*) or BY4742 (*Matα*, *leu2*Δ*0*, *lys2*Δ*0*, *ura3*Δ*0*, *his3*Δ*1*). Strains AJ001 (BY4741, *sdo1*Δ::*HIS3*), WK001 (BY4741, *por1*Δ::*KanMX4*, *sdo1*Δ::*HIS3*), and LJ109 (BY4741 rho^0^) have been described previously [32, 53]. Deletion strains for *DBP3*, *DBP7*, *DOM34*, and *YAR1* were obtained from Open Biosystems (Layafette, CO, USA). Cells were cultured at 30 ˚C on enriched yeast extract, peptone based medium supplemented with 2% glucose (YPD) [80]. For iron depleted conditions medium was adjusted to pH 6.0 with 3-morpholinopropanesulfonic acid (MOPS) and 40 µM or 120 µM BPS was added to YPD broth or solid medium, respectively. Yeast transformations were performed using the lithium acetate procedure [81] and transformants were selected using synthetic complete (SC) medium lacking uracil. Promoter-*lacZ* reporter plasmids pLJ519 (*ACT1*-*lacZ*) and pLJ440 (*FET3*-*lacZ*) have been previously described [82, 83].

### Measurement of intracellular iron and ROS levels

Cells were cultured overnight and were collected at OD600 of approximately 1. Cell pellets were washed with Tris HCl with EDTA, pH 6.5 and then Type I water. Intracellular iron was measured using graphite furnace atomic absorption spectroscopy (PerkinElmer, USA). The iron concentration was reported as nmole Fe/10^9^ cells. To measure intracellular ROS levels, cells at final OD600 of approximately 1 were incubated with 10 μM 2,7-dichorofluorescein diacetate (H_2_DCFDA) (Sigma, U.S.A.) for one hour. Cells were lysed and the fluorescence signal was measured as described previously [84]. Fluorescence intensity was normalized to the protein concentration of each sample.

### Protein carbonylation analysis and immunoblots

Carbonylated proteins were detected following derivatization with 2,4-dinitrophenylhydrazine (DNPH) [85]. Lysates were prepared using the glass bead homogenization in 10 mM NaPO_4_ pH 7.8, 1% Triton X-100, 5 mM EDTA pH 8, and 50 mM NaCl from cells cultured in YPD medium alone or treated with 2 mmol/L H_2_O_2_ for 1 hour. Whole cell lysates from each sample were reacted with DNPH for 15 minutes at room temperature and were resolved on denaturing polyacrylamide gels. DNP-derivatized proteins were detected with an anti-DNP antibody (Merck Millipore, USA) at a 1:5000 dilution. Sod2p protein was detected using anti-Sod2p antibody (JH633) provided by Dr. Valeria Culotta (Johns Hopkins University, USA) at a dilution of 1:5000 [86]. Pgk1p abundance was used in each experiment as loading control with an anti-Pgk1p antibody (Abcam, USA) at a 1:5000 dilution. Anti-mouse and anti-rabbit HRP conjugated secondary antibodies were utilized at a 1:10,000 dilution for ECL detection (Merck Millipore, USA). A G:Box Chemi XL1.4 chemiluminescence imaging system (Syngene, UK) was used for imaging of immunoblots. Quantitation was performed using ImageJ 1.45S software [87].

### Biochemical assays

β-galactosidase assays utilized transformants containing either the *FET3*-*lacZ* or *ACT1*-*lacZ* reporters grown in synthetic media lacking uracil. Cell lysates were prepared using the glass bead homogenization procedure [88] and activity was assayed with, o-nitrophenyl-beta-D-galactopyranoside (ONPG), in 60 mM Na_2_HPO_4_, 40 mM NaH_2_PO_4_, 10 mM KCl, 1 mM MgSO_4_, 1 mM phenylmethylsulfonyl fluoride, and 1 mM dithiothreitiol. The results were assayed at least in duplicate and were reported in Miller units [89].

Superoxide dismutase activity assays were performed using yeast cells grown in YPD to a final OD600 of approximately 1. Cell extracts were prepared and protein content of whole cell lysates was measured by Bradford assay [90]. SOD activity was analyzed by non-denaturing gel electrophoresis and staining with nitro blue tetrazolium (NBT) as previously described [39, 91]

Aconitase and succinate dehydrogensase activity assays were performed according to published methods [52, 92, 93]. Briefly, cells were grown in YPD medium for 40 hours, reaching stationary phase. For analysis of aconitase activity, cells were lysed using glass bead homogenization in KH_2_PO_4_ (pH 7.4) with 1 mM phenylmethylsulfonyl fluoride. The activity of aconitase was monitored in 20 mM Tris-HCl pH 7.4, 100 mM NaCl, and 0.5 mM *cis*-aconitate following the conversion of cis-aconitate to isocitrate at 240 nm using a UV-Vis Spectrophotometer (UV-2600) (Shimadzu, Japan). Crude mitochondria for analysis of SDH activity were isolated from cell lysates prepared with glass homogenization in 0.6 M sorbitol, 10 mM HEPES (pH 7.4) containing 1 mM PMSF using differential centrifugation as previously described [94]. SDH activity was monitored in 50 mM HEPES (pH 7.4), 0.1 mM EDTA, 1 mM KCN, 100 µM phenazine methosulfate, and 20 mM succinate following the reduction of dichlorophenol indophenol (5 µM) at 600 nm using a Genesys 20 Spectrophotometer (Thermo Spectronic, USA).

### Statistical analysis

All data are presented as mean + standard deviation. One-way ANOVA with post-hoc Tukey test or T-test was used to determine statistical significance * P < 0.05, **P < 0.01, and ***P < 0.001.

## Data Availability

The datasets used and/or analyzed in the current study are available from the corresponding author upon request.

## Abbreviations

SDS: Shwachman-Diamond syndrome
ISC: Iron-sulfur cluster
VDAC: Voltage-dependent anion channel
ER: Endoplasmic reticulum
BPS: Bathophenanthroline disulfonic acid
ROS: Reactive oxygen species
